# The effect of parental rearing conditions on offspring life history in *Anopheles stephensi*

**DOI:** 10.1186/1475-2875-6-130

**Published:** 2007-09-24

**Authors:** Katrina Grech, Liam Aye Maung, Andrew F Read

**Affiliations:** 1 Institutes of Evolution, Immunology and Infection Research School of Biological Sciences, Ashworth Laboratories, The University of Edinburgh, Edinburgh EH9 3JT, UK; 2 Epidemiology and Population Biology, Pentlands Science Park, Bush Loan, Moredun Research Institute, Penicuik, EH26 0PZ, UK

## Abstract

**Background:**

The environmental conditions experienced by parents are increasingly recognized to impact the success of offspring. Little is known on the presence of such parental effects in *Anopheles*. If present, parental effects could influence mosquito breeding programmes, some malaria control measures and have epidemiological and evolutionary consequences.

**Methods:**

The presence of parental effects on offspring emergence time, size, survival, blood meal size and fecundity in laboratory reared *An. stephensi *were tested.

**Results:**

Parental rearing conditions did not influence the time taken for offspring to emerge, or their size or survival as adults. However, parental effects were influential in determining the fecundity of daughters. Counter-intuitively, daughters of parents reared in low food conditions produced larger egg clutches than daughters of parents reared in high food conditions. Offspring reared in low food conditions took larger blood meals if their parents had also experienced a low food environment.

**Conclusion:**

So far as we are aware, this is the first evidence of parental effects on progeny in *Anopheles*.

## Background

The reproductive success of an individual is dependent on genetic background, environmental conditions, and interactions between these. One factor which is increasingly recognized to have a profound impact on individual success is the environmental conditions experienced by their parents [1-4] Parental effects have been demonstrated in a wide range of taxa [e.g. [5-18]]. Some of these effects arise from environmental constraints where, for instance, nutrient deprivation in mothers results in less well-provisioned and hence smaller offspring. Parental effects can also be adaptive if parents can perceive cues in their environment and adjust per offspring investment so as to optimize offspring fitness. For instance, in the freshwater crustacean *Daphnia*, mothers kept in poor conditions alter offspring size, survival, fecundity or resistance to parasites [e.g. [16,19,20]]. Here, mothers in poor environments increase per offspring investment in the few offspring they can produce. Thus, there are various routes by which maternal effects can affect important offspring traits such as development time, survival and fecundity.

Anopheline mosquitoes (Diptera: Culicidae) are medically important vectors, responsible for the transmission of many diseases including malaria, filariasis and several arboviral diseases. The distribution of *Anopheles *mosquitoes is an important factor in determining the prevalence of *Plasmodium *infections and is influenced by the presence of suitable blood-meal hosts and oviposition sites. Female preference studies indicate that fecund mosquitoes choose oviposition sites based on many factors including the presence/absence of conspecific instars (which may indicate high food levels), food sources, moisture content and the presence/absence of potential predators [e.g. [21-25]]. Such behaviour indicates that female mosquitoes have the ability to alter their offspring fitness through behaviour and raises the question of whether *Anopheles *parents may also alter investment in their offspring in response to their own condition, or the environmental conditions their offspring might experience.

Despite the hope for large scale rearing and release of sterile males and genetically modified mosquitoes for malaria control, little is known about the effect of *Anopheles *larval parental rearing experience on offspring success. Yet, central to the success of any breeding and release strategy is the production of large numbers of laboratory-reared mosquitoes, and the fitness of released mosquitoes [26,27]. Moreover, many larval control programmes are aimed at actively degrading larval habitats, with unknown effects on the fitness of survivors and their progeny.

To begin the study of non-behavioural parental effects on *Anopheles *life-history, a laboratory study was conducted where *Anopheles *larvae experienced either the same or different rearing conditions to that experienced by their parents (high or low food availability). After varying the parental environment, the offspring fitness components of emergence time, size, survival, blood meal size and fecundity were measured. As genetic and phenotypic variance is often greater in stressful environments, particular attention is paid to parental effects in offspring reared in low food environments[5,28,29]. This study shows that parental rearing conditions can influence offspring life history.

## Methods

### Experimental design

**Figure 1:**
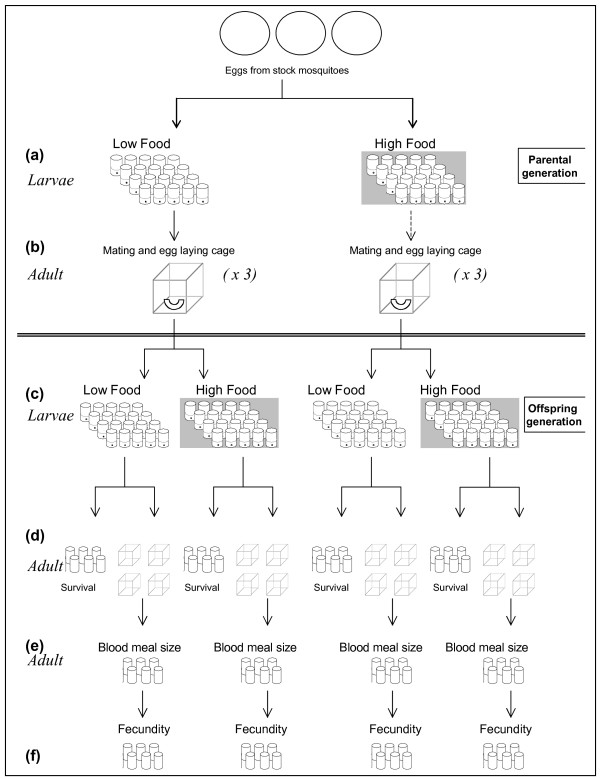
The experimental set up. (a) In experiments 1 and 2, a total of 320 larvae were separated into the parental treatments of high and low food, with individual larvae placed in a standard 30 ml vial containing 5 mls of distilled water. (b) After emergence, adult parents were transferred to a mating cage and kept in good conditions. (c) Eggs from within treatment matings were split into high and low food. (d) In experiment 1, post emergence, the offspring were starved and their survival determined. In experiment 2, half of each offspring treatment was starved, while the other half was transferred to one of four mating cages for a blood meal. (e) After the blood meal individual females were transferred to standard vials for haematin collection. (f) After haematin collection individual females were transferred to standard vials for egg laying.

Mosquitoes originated from a longstanding laboratory stock of *An. stephensi *and were reared under standard laboratory conditions at 27 ± 2°C, 70% humidity and in a 12:12 light: dark cycle. Eggs were hatched in three plastic trays (25 cm × 25 cm) filled with 1.5 L of distilled water. Two days after hatching, larvae from the three trays were mixed and 200 transferred to 30 ml vials containing 5 mls of distilled water, where they were reared individually (Figure 1a). Half of these larvae were then randomly allocated to the low food treatment group (1 mg of Tetrafin^® ^food per day) and half to the high food treatment group (10 mg of Tetrafin^® ^food per day). When individuals pupated, their vial was covered with fine nylon gauze until emergence. Food levels were determined from an earlier pilot study and include the range where 100% emergence occurred. The position of the vials within the insectary was fully randomized at this and all subsequent stages.

On emergence, the mosquitoes were pooled into three mesh cages (30 × 30 × 30 cm) per treatments groups and provided with 10% glucose solution *ad libitum *(Figure 1b). Approximately one week after emergence, one anaesthetized mouse was placed on each cage from which the mosquitoes were allowed to feed for 20 minutes. One day later bowls of water were introduced into the cage for oviposition.

These eggs were then pooled and hatched in six plastic trays for each treatment and 400 individual larvae (200 from each parental treatment group) transferred to a standard vials as above (Figure 1c). The larvae were allocated to either the high or low food treatment groups (as above) and monitored daily for pupation. When individuals pupated, they were transferred to a vial that was covered with fine nylon gauze and maintained without food for survival analysis (Figure 1d). On death, mosquitoes were transferred to a 1 ml tube and refrigerated until the end of the experiment. At the end of the experiment the wings of each mosquito were dissected and measured from the distal to dorsal points using microscopy.

This experiment was repeated eight weeks later. In the repeat experiment, offspring were separated into two groups for either survival analysis as in the previous experiment, or for fecundity analysis after a blood meal (Figure 1d and 1e). Fecundity analysis involved transferring both male and female offspring into mating cages (30 × 30 × 30 cm), with four mating cages per offspring group. Five days after emergence, anaesthetized mice were placed on each of the 16 mating cages and the mosquitoes allowed feed for 20 minutes. These mice had all been infected 14 days before the feed with *Plasmodium chabaudi*, as the original intention was to determine whether vectorial capacity was affected by parental environmental conditions. However, most unexpectedly on the day of the blood-meal, neither asexual nor sexual parasites were present in any of the mice and on dissection of the female mosquitoes after egg-laying, no transmission had occurred. As all infected mice were parasite negative on the day of the feed and were randomly assigned treatment groups which were replicated, any treatment effect should be unrelated to any effect of infection. Immediately after the feed, each female mosquito was transferred to a clean vial covered with fine nylon gauze for three days to allow all haematin (a by-product of decomposition of haemoglobin) to be excreted (Figure 1e), from which blood meal size was estimated [30]. Excreted haematin collected in the bottom of the vial was dissolved in 1 ml of 1% LiC0_3 _solution. The absorbance of the resulting mixture was read at 387 nm in a spectrophotometer using LiC0_3 _solution as a blank and compared with a standard curve made with porcine serum haematin (Sigma Aldrich). Solutions that were within the error range of the LiC0_3 _blanks (absorbance < 0.01) were eliminated from the analysis and classified as non-feeders. After the 3-day haematin collection period, mosquitoes were moved to new 30 ml tubes containing 3 mls of water to allow oviposition (Figure 1f).

### Trait definition

The fitness components of emergence time, survival, adult size, blood meal size and fecundity were measured. Both emergence time and survival were measured as the number of days required for either a) the larvae to emerge as an adult, post hatching, or b) taken to die post emergence. As an indicator of size, the length of one wing per mosquito from the distal to dorsal points using microscopy was measured. Haematin mass was used as an indicator of blood meal size, while fecundity was determined by counting the number of eggs laid over the three days following the blood meal.

### Statistical analysis

The life history traits of emergence time and survival were analysed using Proportional Hazards (JMP in 5.1). Adult size, blood meal size and fecundity were analysed using General Linear Models. The explanatory variables were parental food (two levels), offspring food (two levels), gender (two levels) and where relevant, experimental block (two levels). For all models, a maximal model including all two and three-way interactions was fitted first. Models were then minimized by removing non-significant terms beginning with the highest-level interaction. In no cases were any of the three-way block*parent*offspring condition interactions significant and these are therefore not reported. Significant block*parent or block*offspring interactions did occur, but as they only reflected differences in magnitude between blocks, they are not reported. For the blood meal analysis as well as the fecundity analysis, the average blood meal size per replicate cage and the average number of eggs per replicate cage were used as response variables (n = 16), with average adult size as a covariate. This is a conservative approach to deal with the issue of pseudoreplication of treatments arising from mosquitoes fed on the same mouse. The statistical significance of the main effect of mouse and of the interactions between mouse and the covariates (adult size and blood meal size) was also tested. For both of the response variables of blood meal size and fecundity, the main effect of mouse and the interactions were non-significant.

## Results

### Sample sizes

In experiment 1, two hundred larvae were split into either a high food group or low food group, to form the parental generation. Once adults, these parents were mated within groups (three per treatment) and 400 eggs from the parental high food generation and 400 eggs from the parental low food generation were used for the offspring experiments. Of these, complete records of the emergence time, survival, gender and size were noted for 460 mosquitoes which were included in the analysis. In the repeat experiment, 120 larvae formed the parental generation, from which, 400 eggs from the parental high food generation and 400 eggs from the parental low food generation were used for the offspring analysis. Of these, emergence time was noted for 478 mosquitoes, with 140 of used in the survival trial and 287 used for blood meal and fecundity analysis. Altogether, 149 females were included the fecundity trials in which 19,844 eggs were counted.

### Time taken to emerge

**Figure 2:**
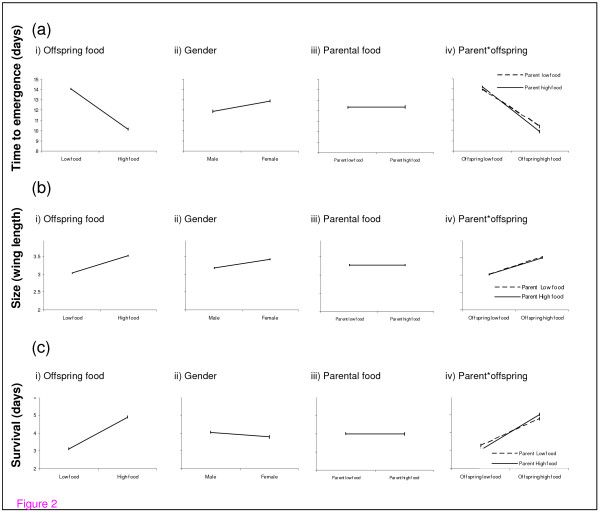
2a: Summary graphs showing the main effect of i) Offspring food (low and high) ii) Gender (male and female) iii) Parental food (low and high) and the iv) interaction between parent and offspring on emergence time. Emergence time was measured as the number of days taken post hatching to emerge as an adult. In total the emergence time of 938 mosquitoes was recorded. For this and all following analysis, 'experimental block' interactions were fitted. Each graph including those below, represents the least square means and the associated standard error. Note that in some cases the dashed line is obscured by the solid line in the interaction graphs. 2b: Summary graphs showing the main effect of i) Offspring food ii) Gender iii) Parental food and the iv) interaction between parent and offspring on adult size. Adult size was determined from the wing length of each mosquito. In total the wing length of 747 mosquitoes was recorded. 2c: Summary graphs showing the main effect of i) Offspring food ii) Gender iii) Parental food, and the iv) interaction between parent and offspring on adult survival. Adult survival was determined as the number of days the mosquito remained alive, post hatching in the absence of water or glucose. In total the wing length of 747 mosquitoes was recorded.

The time taken for larvae to emerge as adults post-hatching was determined by offspring food conditions and to a lesser degree by offspring gender (Figure 2a, Table 1-1). Larvae reared in high food conditions emerged from pupation up to four days earlier than larvae reared in low food conditions and males emerged on average, one day before females. The food levels experienced by the parental generation did not influence emergence time and this lack of parental influence was constant across offspring food levels (Figure 2a, Table 1-1).

**Table 1:** The effects of parent food level, offspring food level and an interaction between them on the fitness components of emergence time, adult size, survival, blood meal size and fecundity. * = <0.05, ** = <0.01, *** = <0.001, **** = <0.0001

	Fitness	Effects	Test Statistic	p	Significance
	component				
1	**Emergence time (days)**				
		Parental food	χ^2 ^= 2.7	0.09	
		Offspring food	χ^2 ^= 699.7	**<0.0001**	***
		Gender	χ^2 ^= 41.4	**<0.0001**	***
		Parental*Offspring	χ^2 ^= 1.2	0.21	

2	**Size (wing length)**				
		Parental food	F_1,593 _= 1.2	0.27	
		Offspring food	F_1,594 _= 2567	**<0.0001**	***
		Gender	F_1,594 _= 900	**<0.0001**	***
		Parental*Offspring	F_1,592 _= 0.7	0.39	

3a	**Survival (days)**				
		Parental food	χ^2 ^= 1.1	0.30	
		Offspring food	χ^2 ^= 96.7	**<0.0001**	***
		Gender	χ^2 ^= 1.9	0.17	
		Parental*Offspring	χ^2 ^= 1.1	0.30	
					
3b	controlling for offspring adult size	Parental food	χ^2 ^= 0.8	0.38	
		Offspring food	χ^2 ^= 62.9	**<0.0001**	***
		Gender	χ^2 ^= 1.6	0.20	
		Parental*Offspring	χ^2 ^= 1.6	0.20	

4a	**Blood meal size (haematin)**				
		Parental food	F_1,13 _= 3.29	0.09	
		Offspring food	F_1,14 _= 7.3	**0.017**	*
		Parental*Offspring	F_1,12 _= 0.8	0.39	
					
4b	controlling for offspring adult size	Parental food	F_1,12 _= 3.1	0.1	
		Offspring food	F_1,13 _= 6.9	**0.02**	*
		Parental*Offspring	F_1,11 _= 0.9	0.35	

5a	**Fecundity (egg number)**				
		Parental food	F_1,14 _= 6.7	**0.02**	*
		Offspring food	F_1,13 _= 3.2	0.096	
		Parental*Offspring	F_1,12 _= 2.5	0.13	
					
5b	controlling for offspring adult size	Parental food	F_1,13 _= 5.9	**0.03**	*
		Offspring food	F_1,12 _= 3.0	0.1	
		Parental*Offspring	F_1,11 _= 2.4	0.14	
					
5c	controlling for daughters blood meal size	Parental food	F_1,11 _= 16.7	**0.0018**	**
		Offspring food	F_1,11 _= 8.0	**0.016**	*
		Parental*Offspring	F_1,11 _= 5.6	**0.037**	*

### Size

Offspring larval food level was also the main factor influencing the size of offspring once they reached adulthood (Fig 2b, Table 1-2). Larvae emerging from high food conditions were on average 16% larger than those emerging from low food conditions. Gender was also an important determinant of offspring size, with females being 7% larger than males (Figure 2b, table 1-2). The larval food levels of the parental generation did not influence offspring adult size (Figure 2b, Table 1-2).

### Survival

The food level experienced by the offspring was again the only factor found to be influencing adult survival (Figure 2c, Table 1-3a). As expected, offspring reared in high food conditions survived for longer than offspring reared in low food conditions. Survival did not differ between the males and the females and was unaffected by parental rearing conditions (Figure 2c, Table 1-3a). Larger offspring survived for longer than smaller offspring (χ^2 ^= 148, df = 1, p = <0.0001), but offspring food level was still a major determinant of survival even when controlling for offspring size (Figure 2c, Table 1-3c).

### Blood feeding

Nineteen out of the 149 females that were given access to a blood meal were classified as non feeders. No effect of parental food level, offspring food level or an interaction between the two influenced female propensity to fed (parent: χ^2 ^= 2.8, df = 1, p = 0.09; offspring: χ^2 ^= 2.7, df = 1, p = 0.1; parent*offspring: χ^2 ^= 2.5, df = 1, p = 0.1, respectively).

**Figure 3:**
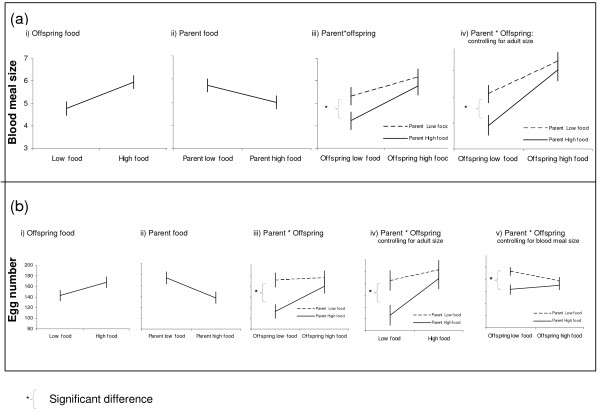
3a: Summary graphs showing the main effects of i) Offspring food and ii) Parental food, as well as iii) the interaction between parent and offspring and iv) the interaction between parent and offspring on blood meal size, controlling for adult size. Haematin concentration was used as an indicator or blood meal size. For each treatment 10–15 female mosquitoes fed on 4 replicate mice. The plotted points are therefore the average blood meal size of each mosquito per mouse (n = 16) and the associated standard error. 3b: Summary graphs showing the main effects of i) Offspring food and ii) Parental food, as well as iii) the interaction between parent and offspring and iv) the interaction between parent and offspring on fecundity, controlling for blood meal size. Fecundity was determined by counting the number of eggs laid over the 3 days following a blood meal. For each treatment 10–15 female mosquitoes fed on 4 replicate mice. The plotted points are therefore the average total number of eggs laid per mosquito per mouse (n = 16) and the associated standard error.

Of the 130 females that did feed, only the level of food they themselves experienced as larvae influenced the size of the blood meal they took as adults (Figure 3a, Table 1-4a). Daughters reared in high food conditions took 25% larger blood meals than the daughters reared at low food conditions. Controlling for adult size, it was again found that blood-meal size was influenced only by the food level experience of the offspring (Figure 3a, table 1-4b). However, genetic and phenotypic variance is often greater in stressful environments [5,28,29]. Indeed, parental effects were apparent when the offspring reared on low food only were considered: blood meal size was influenced by the larval food level of parents, even when controlled for adult size, with the offspring of parents reared in low food conditions taking larger blood meals (parent: F_1,6 _= 27.4, p = 0.002, parent controlled for size: F_1,5 _= 138.3, p < 0.0001, respectively).

### Egg number

Parental effects influenced offspring fecundity. Offspring of parents reared in low food conditions produced more eggs than the offspring from parents reared in high food conditions (Figure 3b, Table 1-5a). Parental food level influenced offspring egg number, even when controlling for offspring size (Figure 3b, Table 1-5b). Considering the offspring reared on low food only, the fecundity of low food daughters was influenced by the larval food level of their parents, even when controlled for adult size (F_1,6 _= 13.6, p = 0.01, F_1,5 _= 12.3, p = 0.017, respectively).

Blood meal size and egg number were positively correlated, with larger blood meal sizes resulting in increased egg production (F_1,14 _= 34.1, p < 0.0001). The number of eggs produced for a given blood meal size was influenced by offspring as well as parental larval food level (Figure 3b, Table 1-5c). Parental effects influenced the fecundity of daughters reared at low food conditions but not of daughters reared at high food (F_1,5 _= 16.1, p = 0.01, F_1,5 _= 4.5, p = 0.09, respectively). For the same size blood meal, the daughters reared at low food conditions were 20% more fecund if their parents had also been reared at low food levels.

## Discussion

This study sought to establish the potential for parental effects to influence offspring reproduction in *Anopheles*. It was found that parental environment had variable effects on mosquito life history traits. Parental effects did not influence the time taken for offspring to emerge, offspring size or survival. These traits were influenced by current offspring food levels only. However, there was a parental effect on blood meal size, but only in offspring reared in the low food environment. Daughters raised in the low food environment took larger blood meals if their parents had also experienced low food than if their parents had experienced high food. It is tempting to think that the enlarged blood meals were to compensate for poor maternal provisioning and resource acquisition during larval development.

Parental effects were also influential in determining the fecundity of their daughters. Daughters from parents reared in low food conditions produced more eggs than daughters from parents reared in high food. This increased fecundity may arise to compensate for expected decreased longevity in low food environments. Although studies showing condition-mediated life history shifts affecting clutch size are limited, shifts to earlier reproduction due to parasite and predator mediated effects have been previously observed in invertebrates [31,32]. For the female mosquitoes in this study, a reduced adult lifespan of even a few days could dramatically decrease total lifetime fecundity. In an environment where numerous bouts of reproduction are not probable, the optimal strategy may be to shift resources into few larger reproductive efforts [33]. If this explanation is correct, there must be costs of increased fecundity, possibly in terms of survival in benign environments or in terms of offspring quality.

Does the potential for adaptive parental provisioning exist in this system? For this to be the case (i) ecologically-relevant environmental variation would need to affect offspring fitness, (ii) parents would need be able to predict their offspring's environment through reliable cues, and (iii) parents would be able to adaptively adjust the phenotype of their offspring to the anticipated environment [15]. Evidence for environmental variation influencing mosquito fitness is abundant. Temperature, humidity, parasitism, sugar feeding and plant extracts have all been found to influence fecundity [e.g. [34-38]]. The potential for prediction and life history adjustment are less clear. Oviposition preference indicates that mothers can assess the environment their larval offspring will inhabit. However, the frequently ephemeral oviposition sites of *Anopheles *may make it difficult for a female mosquito to predict the environment that her offspring will inhabit. With little direct evidence either way; there is much potential for work on adaptive parental effects in *Anopheles*.

The parental effects reported here may be relevant to control strategies against vector-borne diseases. The deliberate ecological manipulation of larval habitats is a mainstay of vector control against malaria [e.g. [39-44]]. Moreover, the large scale releases of captive raised genetically-modified or sterile *Anopheles *are being proposed as possible future malaria control strategies [e.g. [45-50]]. Where maximal fecundity is an aspiration of mass rearing programmes, our finding that fecundity is influenced by parental condition argues for further work to optimize the offspring and parent rearing conditions. Furthermore, if the fecundity effects arise because of adaptive life history provisioning, then there must be fitness costs to larger clutches (otherwise, it would be hard to explain why only the offspring of parents raised in poor conditions respond to poor conditions by increasing fecundity). It may be that maximizing fecundity in mosquito captive rearing programmes would be sub-optimal. In the rearing and release of hatchery salmon, for example, it has been suggested that selection for increased fecundity has resulted in decreased egg size, which was linked to decreased survival [51]. By its nature, captive breeding selects for quantity over quality. It may be that large-scale rearing in benign environments may lead to their maladaptation to wild conditions. These considerations, together with the data reported here, point to the need for more work on parental effects in malaria vectors. Obvious next steps include the analyses of wild caught mosquitoes, of other *Anopheles *species, and of other feeding regimes.

## Authors' contributions

KG designed and analysed the experiments described here and wrote manuscript. LM conducted experiment 1, assisted in the planning of both experiments and the initial analysis. AR assisted in the planning of the experiments, the writing of the manuscript and obtained the funding. All authors read and approved the final manuscript.
